# Effects of virtual reality-based cognitive training in older adults living without and with mild dementia: a pretest–posttest design pilot study

**DOI:** 10.1186/s13104-019-4810-2

**Published:** 2019-11-27

**Authors:** Ludmiła Zając-Lamparska, Monika Wiłkość-Dębczyńska, Adam Wojciechowski, Marta Podhorecka, Anna Polak-Szabela, Łukasz Warchoł, Kornelia Kędziora-Kornatowska, Aleksander Araszkiewicz, Paweł Izdebski

**Affiliations:** 1 Faculty of Psychology, Kazimierz Wielki University, Staffa 1, 85-867 Bydgoszcz, Poland; 2 Institute of Information Technology, Lodz University of Technology, Lodz, Poland; 3 Department of Geriatrics, Collegium Medicum in Bydgoszcz, Nicolaus Copernicus University in Toruń, Toruń, Poland; 4 Department of Psychiatrics, Collegium Medicum in Bydgoszcz, Nicolaus Copernicus University in Toruń, Toruń, Poland

**Keywords:** Cognitive aging, Dementia, Cognitive remediation, Virtual reality, Video games

## Abstract

**Objective:**

Modern technologies are increasingly used in the development of cognitive interventions for older adults. Research into possible applications of virtual reality in such interventions has begun only recently. The aim of present study was to evaluate the effects of 8 sessions of VR-based cognitive training using the GRADYS game in healthy older adults (n = 72; aged 60–88) and older adults living with mild dementia (n = 27; aged 60–89).

**Results:**

Older adults with mild dementia demonstrated worse baseline cognitive performance than participants without dementia. Both groups showed progress in training, which was greater in healthy older adults. There were also significant differences in cognitive functioning before and after the training. However, positive changes were revealed almost exclusively in the group of older adults without dementia. Based on the findings, we can recommend the GRADYS game for cognitive enhancement and as a possible counter-measure for cognitive decline experienced in normal cognitive ageing. Our results provide also support for the usefulness of VR technology in cognitive interventions in older adults. The use of the GRADYS game in persons living with dementia, however, would require several of the hardware and software modifications.

*Trial registration* ISRCTN17613444, date of registration: 10.09.2019. Retrospectively registered

## Introduction

The dynamic development of modern technologies raises questions as to the possibilities of their application in older adults in order to support their cognitive functioning. The efficacy of cognitive training in healthy older adults has been confirmed by meta-analyzes [1–4]. Several types of cognitive interventions [5] have proven beneficial also in older adults diagnosed with mild cognitive impairment (MCI) [6–10] which assumes cognitive functioning at the level between a healthy cognitive ageing and dementia [11]. Studies conducted on participants living with dementia are less clear. Some reviews and meta-analyzes have indicated positive effects of CI in dementia [12, 13], but others have led to opposite conclusions [14, 15]. Furthermore, positive effect on cognitive functioning in dementia is caused by cognitive stimulation, but not by cognitive training [16–18]. Currently, more and more studies on cognitive interventions employ modern technologies such as computerized cognitive training and video games. Meta-analyzes and systematic reviews provide evidence for the efficacy of this type of interventions in healthy older adults [19–23] and persons with MCI [24–27]. In persons with dementia, however, the evidence remains unclear and questionable [24, 28, 29]. A relatively new research area of growing interest in cognitive interventions concerns the use of virtual reality (VR). One of major benefits of VR is that it provides an immersive and naturalistic environment, which might increase the ecological validity of the intervention [30, 31] and allows for training of cognitive skills that are relevant for real-world contexts [32–34]. This can make it easier for persons with dementia to benefit from cognitive training. Nevertheless, there is little research on the efficacy of VR-based cognitive interventions in relation to cognitive aging. In a systematic review of VR applications in healthcare, none of the included studies concerned cognitive interventions in older adults [35]. In a newest systematic review, concerning the efficacy of technology-based cognitive training in persons with MCI, VR was used in two out of 26 studies [26]. In the most recent meta-analysis on the efficacy of VR-based interventions in persons with MCI or dementia, VR-based cognitive training was used in 6 out of 11 studies and only one of them used a fully immersive technology [36]. This meta-analysis indicated a medium effect of VR-based interventions for cognition, larger in participants with MCI than in persons with dementia.

Recently, several studies on the use of VR-based cognitive training in healthy older adults or older adults with MCI have been published [34, 37–40]. However, the study samples were usually small, including case studies, and some articles have described only partial results from initial phases of the study or even only the design of the intervention.

## Main text

### Method

A pretest–posttest study design was applied to evaluate the effects of the VR-based cognitive training using the GRADYS game in older adults without and with mild dementia (Additional file 1: Figure S1, Additional file 2).

The exclusion criteria from the sample included the presence of mental disorders and serious somatic illnesses, as well as the presence of visual, auditory and motor impairments that could prevent the use of the game. All these criteria have been checked in a structured interview with the participant and verified by the care-taker in the case of participants with dementia. The initial sample comprised 150 participants, 75 in each group. The complete data were obtained from 99 participants aged 60–89, including 72 healthy older adults (54 women, age: *M *= 67.86, *SD *= 5.83; years of education: *M *= 13.61, *SD *= 3.86; Mini Mental State Examination (MMSE): *M *= 28.69, *SD *= 1.22) and 27 older adults with mild dementia (22 women, age: *M *= 72.04, *SD *= 7.43; years of education: *M *= 12.58 *SD *= 3.33; MMSE: *M *= 22.33, *SD *= 1.21). Participants who withdrew from the study, ceased their participation before the 3rd training session. A significant dropout of participants with mild dementia were mainly due to difficulties in understanding and coordinating the game control interface.

The administered intervention was based on the GRADYS game, which is a VR-based cognitive training containing four modules: attention, memory, language, and visuospatial processing. The storyline of each module scenario consists of tasks inspired by daily life. Each module has three difficulty levels. The game software included also a tutorial module to help participants to learn how to operate on the game interface. The game was controlled with the Oculus Rift DK2 and the Xbox 6DOF control pad.

Both study groups underwent eight individual training sessions, two per week. Each session consisted of two game modules: memory and attention or language and visuospatial processing. A single session lasted from 45 min to an hour. In each module the participants started at the lowest difficulty level and moved to a higher level in the next session having reached 75% accuracy in the previous one. Similarly, in the case of accuracy falling below 50%, the participants returned to a lower difficulty level. The participants were accompanied by a training assistant throughout the whole session.

Two sets of measures were used:

A. Screening tests: a structured interview; the Mini Mental State Examination (MMSE) [41]; the Addenbrooke Cognitive Examination III (ACE-III) [42].
B. Measures of cognitive abilities according to four modules (in pretest and posttest; AV stands for alternative versions of the test used in pretest and posttest, for other tests the same version was used in both pretest and posttest):
  1. Attention: the Digit Symbol test from WAIS-R (PL) [43]; the Colour trail test (CTT)—Adult version [44] (AV), the d2 Test of Attention (indices: WZ—speed of processing, %B—percentage of errors, WZ-B—error corrected speed of processing, ZK—ability to concentrate) [45];
  2. Memory: the Digit Span test from WAIS-R (PL) [43], the Benton Visual Retention Test (BVRT) [46] (AV), the Rey Auditory Verbal Learning Test (AVLT) [47] (AV), the Famous Faces Test [47] (AV), the Rey–Osterrieth complex figure test (ROCF)—delayed reproduction [47] (AV);
  3. Language: the Verbal Fluency from ACE-III [42]; the Boston Naming Test (BNT) [47];
  4. Visuospatial processing: the Block Design test from WAIS-R (PL) [43], the Rey–Osterrieth complex figure test (ROCF)—direct copying [47] (AV).

### Results

Better baseline cognitive performance was observed in the group of healthy older adults in general (Hotelling *T*^2^ = 130.868; *p* < 0.001) and in the majority of cognitive measures (Table 1). In a few of the cognitive measures, no significant differences between groups were observed. Two performance indices in the sustained attention task (d2) were significantly better in the group of older adults with dementia.

**Table 1:** A comparison of baseline cognitive performance in two research groups

Cognitive measures	The Student’s *t* test/the Welch’s *t* test^b^			The Mann–Whitney *U* test^a^	
	*t*^a^	*p*	Hedges’s *g*	*Z* corr.^c^	*p*
Digit symbol WAIS-R (PL)	2.899	0.005	0.65	2.748	0.006
CTT-1 time	− 2.301^d^	0.029	0.76	− 2.986	0.003
CTT-1 errors	− 1.185^d^	0.244	0.32	− 1.427	0.153
CTT-2 time	− 2.361^d^	0.025	0.71	− 3.041	0.002
CTT-2 errors	− 1.457^d^	0.156	0.45	− 2.178	0.029
d2 WZ	− 2.848	0.005	0.68	− 2.829	0.005
d2%B	1.021^d^	0.474	0.16	− 0.519	0.604
d2 WZ-B	− 2.396	0.019	0.54	− 2.675	0.007
d2 ZK	1.014	0.310	0.23	1.226	0.220
Block design WAIS-R (PL)	3.018	0.003	0.68	2.426	0.015
ROCF copy	2.332^d^	0.026	0.63	2.759	0.006
ROCF delayed recall	3.727	< 0.001	0.83	3.502	< 0.001
Digit span—forward WAIS-R (PL)	0.255	0.799	0.06	0.619	0.536
Digit span—backward WAIS-R (PL)	2.683	0.009	0.60	2.426	0.015
AVLT list A, trial 1	2.579	0.011	0.58	2.319	0.020
AVLT list A, trial 5	5.528	< 0.001	1.24	4.558	< 0.001
AVLT list B	3.324^d^	0.001	0.57	3.042	0.002
AVLT list A, trial 6	4.414	< 0.001	0.99	3.949	< 0.001
AVLT list A, trial 7	4.961	< 0.001	1.11	4.206	< 0.001
AVLT list A, recognition	0.221^d^	0.827	0.07	1.729	0.084
BVRT correct reproductions indicator	3.333	0.001	0.75	2.873	0.004
BVRT errors indicator	− 3.642^d^	< 0.001	0.95	− 3.398	< 0.001
Famous faces test	4.778^d^	< 0.001	1.28	4.713	< 0.001
Verbal fluency (ACE-III)	3.967	< 0.001	0.89	3.192	0.001
Boston naming test	2.163^d^	0.038	0.63	1.958	0.050

The means and standard deviations for all the above mentioned cognitive measures in both research groups are presented in Table 3 which contains the means and standard deviations for both groups in pretest and posttest

^a^The t test was supplemented by the non-parametric Mann–Whitney *U* test due to unequal number of participants in both groups

^b^If the Levene’s test indicated the unequality of variances, the Welch’s *t* test was calculated

^c^Correction for ties in the ranking

^d^The Welch’s *t* test

Both groups demonstrated progress throughout the training (the Friedman’s test: *p* < 0.001 for each module in both groups). Yet the group of older adults without dementia showed larger gain in the majority of cognitive modules, except the attention module. Differences between the groups in terms of the maximum difficulty level reached until the last training session, analysed by the Mann–Whitney U test, were significant with regard to memory (*p* = 0.002), visuospatial processing (*p* < 0.001), and language (*p* = 0.006), but not attention (*p* = 0.053).

The results of repeated measures multivariate analysis of variance (RM MANOVA), supplemented by one-way tests, indicated improvement after the training, but mainly in the group of healthy participants (Tables 2 and 3).

**Table 2:** Differences in cognitive performance between pretest and posttest: repeated measures multivariate analysis of variance

Group	Effect	Value	*F*	*p* level	Partial Eta squared	Observed power
Both	Training					
	Wilks’ Lambda	0.613	1.840	0.023	0.39	0.97
	Pillai’s trace	0.387				
	Hotelling’s trace	0.630				
	Training*group					
	Wilks’ Lambda	0.652	1.562	0.073	0.35	0.93
	Pillai’s trace	0.349				
	Hotelling’s trace	0.535				
HOA	Training					
	Wilks’ Lambda	0.326	3.879	< 0.001	0.67	1.00
	Pillai’s trace	0.674				
	Hotelling’s trace	2.063				
MD	Training					
	Wilks’ Lambda	0.022	3.565	0.242	0.98	0.21
	Pillai’s trace	0.978				
	Hotelling’s trace	44.557				

*HOA* healthy older adults, *MD* older adults with mild dementia

**Table 3:** Differences between pretest and posttest for particular cognitive measures: one-way tests computed separately in both groups

Cognitive measures	Groups	Pretest *M (SD)*	Posttest *M (SD)*	Fisher’s *F*	*p* value	*Partial eta squared*
Digit symbol WAIS-R (PL)	HOA	39.917 (13.883)	41.859 (10.801)	3.45	0.067	0.046
	MD	30.962 (13.347)	32.067 (13.904)	1.183	0.286	0.044
CTT-1 time	HOA	67.389 (27.690)	61.930 (22.843)	2.952	0.090	0.04
	MD	105.667 (84.748)	88.080 (40.528)	1.318	0.261	0.048
CTT-1 errors	HOA	0.097 (0.342)	0.028 (0.166)	2.816	0.098	0.038
	MD	0.222 (0.506)	0.040 (0.192)	2.933	0.099	0.101
CTT-2 time	HOA	130.014 (51.600)	120.761 (45.302)	5.167	0.026	0.068
	MD	180.815 (107.236)	193.960 (122.325)	1.67	0.208	0.06
CTT-2 errors	HOA	0.222 (0.717)	0.211 (0.579)	0.017	0.898	0.0002
	MD	0.704 (1.660)	0.520 (1.244)	0.896	0.352	0.033
d2 WZ	HOA	316.493 (125.297)	350.901 (143.112)	18.287	< .001	0.205
	MD	392.958 (99.711)	387.318 (118.466)	0.162	0.691	0.006
d2%B	HOA	23.870 (34.578)	15.273 (14.907)	5.838	0.018	0.076
	MD	18.945 (13.410)	16.299 (11.794)	4.364	0.047	0.144
d2 WZ-B	HOA	256.155 (126.183)	300.167 (134.440)	42.946	< .001	0.377
	MD	320.708 (98.540)	328.00 (116.011)	0.382	0.542	0.014
d2 ZK	HOA	111.329 (47.701)	129.690 (50.946)	14.674	< .001	0.171
	MD	100.375 (48.374)	105.903 (48.714)	1.005	0.325	0.047
Block design WAIS-R (PL)	HOA	21.833 (8.454)	24.183 (8.118)	12.509	< .001	0.15
	MD	15.852 (9.623)	17.017 (9.433)	2.433	0.131	0.086
ROCF copy	HOA	33.028 (4.663)	33.809 (2.893)	1.804	0.184	0.025
	MD	29.596 (7.094)	32.562 (4.878)	8.867	0.006	0.254
ROCF delayed recall	HOA	19.146 (8.329)	21.735 (7.233)	9.795	0.003	0.121
	MD	12.481 (6.693)	12.945 (6.811)	0.216	0.646	0.008
Digit span—forward WAIS-R (PL)	HOA	5.693 (1.931)	5.704 (1.731)	0.21	0.648	0.003
	MD	5.519 (2.471)	5.269 (2.176)	0.651	0.427	0.024
Digit span—backward WAIS-R (PL)	HOA	5.042 (1.699)	5.479 (1.635)	6.619	0.012	0.085
	MD	3.963 (1.190)	3.769 (1.219)	0.435	0.515	0.016
AVLT list A, trial 1	HOA	5.268 (1.784)	5.409 (1.553)	0.353	0.555	0.005
	MD	4.259 (1.583)	4.346 (1.413)	0.089	0.767	0.003
AVLT list A, trial 5	HOA	11.437 (2.354)	11.310 (2.481)	0.268	0.606	0.004
	MD	8.407 (2.620)	8.692 (2.126)	0.694	0.412	0.026
AVLT list B	HOA	5.113 (2.140)	4.634 (2.050)	2.848	0.096	0.039
	MD	4.000 (1.144)	3.962 (1.285)	0.027	0.871	0.001
AVLT list A, trial 6	HOA	9.070 (3.069)	9.366 (3.181)	0.894	0.348	0.012
	MD	6.037 (2.981)	6.500 (3.153)	0.623	0.437	0.023
AVLT list A, trial 7	HOA	9.254 (3.066)	8.873 (3.801)	1.036	0.312	0.014
	MD	5.704 (3.440)	6.154 (3.371)	1.013	0.323	0.038
AVLT list A, recognition	HOA	14.056 (1.727)	14.056 (1.694)	0.000	1.000	0.000
	MD	13.852 (4.688)	13.360 (4.419)	0.272	0.607	0.01
BVRT correct reproductions indicator	HOA	5.889 (1.781)	6.189 (1.962)	2.457	0.121	0.033
	MD	4.444 (2.259)	3.692 (1.957)	5.17	0.031	0.166
BVRT errors indicator	HOA	6.292 (3.009)	5.455 (3.125)	7.234	0.009	0.092
	MD	9.519 (4.219)	9.885 (4.585)	0.221	0.643	0.008
Famous faces test	HOA	9.583 (1.432)	9.471 (1.774)	0.544	0.463	0.008
	MD	7.444 (2.154)	7.600 (2.465)	0.154	0.698	0.006
Verbal fluency (ACE-III)	HOA	10.958 (2.236)	11.343 (2.426)	2.576	0.113	0.035
	MD	8.778 (2.913)	8.630 (3.628)	0.07	0.794	0.003
Boston naming test	HOA	57.722 (3.027)	58.000 (2.974)	1.71	0.195	0.024
	MD	55.259 (5.620)	55.577 (5.500)	0.513	0.48	0.019

*HOA* healthy older adults, *MD* older adults with mild dementia

The RM MANOVA with group as a between-subject variable (Table 2) revealed a significant pre-post training difference in cognitive functioning. The interaction effect was not significant. However, the probability value was only slightly above the assumed value of statistical significance (*α* = 0.05), while partial eta squared indicated a large effect size [48], which suggested that the group may be a factor moderating the effect of training with regard at least to some cognitive measures.

According to RM MANOVA computed for each group separately (Table 2), significant changes occurred only in participants without dementia. In the group of older adults with mild dementia the changes were not significant, however the observed power was low, in turn the effect size was high. It suggests that the statistical power of the test was insufficient to estimate the changes in this group with possible reasons being, at least partially, too small a sample size and a high sampling error.

In turn, one-way tests for the pretest–posttest differences in particular cognitive measures indicated several significant changes in the group of older adults with mild dementia. However, much fewer than in the group of healthy participants (Table 3).

### Discussion

The results lead to a general conclusion that the GRADYS game may be effective cognitive intervention in older adults without dementia. However, the usefulness of the current version of the GRADYS game in persons with mild dementia is questionable.

As expected, before training, participants with mild dementia demonstrated lower cognitive performance than healthy older adults. In a few relatively easy cognitive measures no significant group differences were observed. Interestingly, two performance indices of d2 were significantly better in the group of older adults with mild dementia (WZ, WZ-B). The WZ indicator, however, is vulnerable to overestimation due to the skipping a part of the characters in the row, which was frequently observed in participants with dementia. The value of WZ-B, is a derivative of the WZ value.

Both groups showed progress in the course of training. Nevertheless, among older adults with mild dementia not only less progress, but also a there was a large withdrawal of participants from the sample. Therefore, the results obtained in the group of persons with mild dementia are less reliable and should be interpreted with caution.

Regarding the effects of the training on cognitive functions beyond the game environment, a significant difference in cognitive tests performance before and after training yielded in RM MANOVA suggests a positive impact of the training. At the same time, one-way tests revealed that positive changes were observed almost exclusively in older adults without dementia. Healthy older adults also improved in visuospatial processing, visual aspects of memory and working memory, but not in verbal learning and language. In contrast, in the group of older adults with mild dementia positive changes were observed only in the percentage of errors in the d2 test and in the copying task of ROCF, which belong to the easiest tasks used in the assessment. In this group also a negative cognitive change was observed (in BVRT).

Poor training effects in participants with mild dementia, despite its efficacy in healthy older adults are consistent with the results of previous studies, which have reported that in persons with dementia cognitive training is less effective [16–18]. We hoped that a near-natural VR environment would enhance training efficacy in this group, but we did not observe it.

## Limitations

- The lack of a control group makes it difficult to isolate the training-related effects from the repeated measurement and practice effects. However, in order to reduce the likelihood of the practice effect occurrence in the majority of cognitive measures applied in cognitive assessment, alternative versions of the test were used in pretest and posttest (except for those, where no such versions exist). In addition, because the improvement occurred for some cognitive measures using different versions in the pretest and posttest, and at the same time the improvement did not occur for some measures using the same versions in the pretest and posttest, we conclude that the observed improvement cannot be attributed to the simple practice effect. Further development of the GRADYS game requires evidence of its efficacy obtained in randomised controlled trial.
- Experiencing significant difficulties in game control due to an excessive cognitive burden in using new technology, older adults with mild dementia, were often unable to concentrate on cognitive tasks. To operate the game interface participants had to memorize and coordinate the functions of several buttons on the pad under the right and left thumbs as well as several ways of controlling the game with the Oculus. Additionally, the Oculus made it impossible for participants to visually verify their choice of buttons on the pad, so they had to maintain not only the functional but also spatial mapping in their working memory at all time. Interface control was difficult to achieve in participants with mild dementia even when they were provided with a practice tutorials. Thus, the cognitive burden of game control might have led to low learnability and caused a decreased efficacy of the training in older adults with mild dementia and their withdrawal from training. A significant dropout of participants with mild dementia due to the mentioned game control difficulties limits the conclusions to individuals with mild dementia who maintain a relatively high overall cognitive performance that allows them to understand and coordinate the game control interface. Further development of the game would requires making the game control more natural and easier.

## Supplementary information

**Additional file 1: Figure S1.** The structure of the intervention.

**Additional file 2.** Dataset.

## Data Availability

The dataset is available as a Additional file for the manuscript.

## Abbreviations

ACE-III: Addenbrooke Cognitive Examination III
AD: Alzheimer’s disease
AV: alternative versions (alternative versions of cognitive tests used in the pretest and posttest)
AVLT: Rey Auditory Verbal Learning Test
BVRT: Benton Visual Retention Test
CTT: colour trail test
MCI: mild cognitive impairment
MMSE: Mini Mental State Examination
RM MANOVA: repeated measures multivariate analysis of variance
ROCF: Rey–Osterrieth complex figure test
VR: virtual reality
WAIS-R (PL): Wechsler adult intelligence scale-revised (Polish)

## References

[1] Chiu H-L, Chu H, Tsai J-C (2017). The effect of cognitive-based training for the healthy older people: a meta-analysis of randomized controlled trials. PLoS ONE.

[2] Eschen A (2012). The contributions of cognitive trainings to the stability of cognitive, everyday, and brain functioning across adulthood. GeroPsych J Gerontopsychol Geriatr Psychiatry.

[3] Karbach J, Verhaeghen P (2014). Making working memory work: a meta-analysis of executive-control and working memory training in older adults. Psychol Sci.

[4] Soveri A, Antfolk J, Karlsson L, Salo B, Laine M (2017). Working memory training revisited: a multi-level meta-analysis of n-back training studies. Psychon Bull Rev.

[5] Alves J, Magalhães R, Machado Á, Gonçalves ÓF, Sampaio A, Petrosyan A (2013). Non-pharmacological cognitive intervention for aging and dementia: current perspectives. World J Clin Cases.

[6] Mewborn CM, Lindbergh CA, Stephen Miller L (2017). Cognitive interventions for cognitively healthy, mildly impaired, and mixed samples of older adults: a systematic review and meta-analysis of randomized-controlled trials. Neuropsychol Rev.

[7] Sherman DS, Mauser J, Nuno M, Sherzai D (2017). The efficacy of cognitive intervention in mild cognitive impairment (MCI): a meta-analysis of outcomes on neuropsychological measures. Neuropsychol Rev.

[8] Teixeira CVL, Gobbi LTB, Corazza DI, Stella F, Costa JLR, Gobbi S (2012). Non-pharmacological interventions on cognitive functions in older people with mild cognitive impairment (MCI). Arch Gerontol Geriatr.

[9] Gates N, Sachdev P, Singh MAF, Valenzuela M (2011). Cognitive and memory training in adults at risk of dementia: a systematic review. BMC Geriatr.

[10] Jean L, Bergeron MÈ, Thivierge S, Simard M (2010). Cognitive intervention programs for individuals with mild cognitive impairment: systematic review of the literature. Am J Geriatr Psychiatry.

[11] Roberts R, Knopman DS (2013). Classification and epidemiology of MCI. Clin Geriatr Med.

[12] Ballard C, Khan Z, Clack H, Corbett A (2011). Nonpharmacological treatment of Alzheimer disease. Can J Psychiatry.

[13] Sitzer DI, Twamley EW, Jeste DV (2006). Cognitive training in Alzheimer’s disease: a meta-analysis of the literature. Acta Psychiatr Scand.

[14] Kurz AF, Leucht S, Lautenschlager NT (2011). The clinical significance of cognition-focused interventions for cognitively impaired older adults: a systematic review of randomized controlled trials. Int Psychogeriatr.

[15] Bahar-Fuchs A, Clare L, Woods B (2013). Cognitive training and cognitive rehabilitation for persons with mild to moderate dementia of the Alzheimer’s or vascular type: a review. Alzheimers Res Ther.

[16] Buschert V, Bokde ALW, Hampel H (2010). Cognitive intervention in Alzheimer disease. Nat Rev Neurol.

[17] Huntley JD, Gould RL, Liu K, Smith M, Howard RJ (2015). Do cognitive interventions improve general cognition in dementia? A meta-analysis and meta-regression. BMJ Open.

[18] Spector A, Orrell M, Hall L (2012). Systematic review of neuropsychological outcomes in dementia from cognition-based psychological interventions. Dement Geriatr Cogn Disord.

[19] Kueider AM, Parisi JM, Gross AL, Rebok GW (2012). Computerized cognitive training with older adults: a systematic review. PLoS ONE.

[20] Lampit A, Hallock H, Valenzuela M (2014). Computerized cognitive training in cognitively healthy older adults: a systematic review and meta-analysis of effect modifiers. PLoS Med.

[21] Tetlow AM, Edwards JD (2017). Systematic literature review and meta-analysis of commercially available computerized cognitive training among older adults. J Cogn Enhanc.

[22] Shah TM, Weinborn M, Verdile G, Sohrabi HR, Martins RN (2017). Enhancing cognitive functioning in healthly older adults: a systematic review of the clinical significance of commercially available computerized cognitive training in preventing cognitive decline. Neuropsychol Rev.

[23] Toril P, Reales JM, Ballesteros S (2014). Video game training enhances cognition of older adults: a meta-analytic study. Psychol Aging.

[24] Hill NTM, Mowszowski L, Naismith SL, Chadwick VL, Valenzuela M, Lampit A (2017). Computerized cognitive training in older adults with mild cognitive impairment or dementia: a systematic review and meta-analysis. Am J Psychiatry.

[25] O’Shea DM, De Wit L, Smith GE (2019). Doctor, should i use computer games to prevent dementia?. Clin Gerontol.

[26] Ge S, Zhu Z, Wu B, McConnell ES (2018). Technology-based cognitive training and rehabilitation interventions for individuals with mild cognitive impairment: a systematic review. BMC Geriatr.

[27] Coyle H, Traynor V, Solowij N (2015). Computerized and virtual reality cognitive training for individuals at high risk of cognitive decline: systematic review of the literature. Am J Geriatr Psychiatry.

[28] García-Casal JA, Loizeau A, Csipke E, Franco-Martín M, Perea-Bartolomé MV, Orrell M (2016). Computer-based cognitive interventions for people living with dementia: a systematic literature review and meta-analysis. Aging Ment Health.

[29] Sood P, Kletzel SL, Krishnan S (2019). Nonimmersive brain gaming for older adults with cognitive impairment: a scoping review. Gerontologist.

[30] García-Betances RI, Cabrera-Umpiérrez MF, Arredondo MT (2017). Computerized neurocognitive interventions in the context of the brain training controversy. Rev Neurosci.

[31] Schuchat J, Ouellet É, Moffat N, Belleville S (2012). Opportunities for virtual reality in cognitive training with persons with mild cognitive impairment or Alzheimer’s disease. Non-Pharmalogical Ther Dement.

[32] Negut A, Matu S-A, Sava FA, David D (2016). Virtual reality measures in neuropsychological assessment: a meta-analytic review. Clin Neuropsychol.

[33] Zygouris S, Ntovas K, Giakoumis D (2017). A preliminary study on the feasibility of using a virtual reality cognitive training application for remote detection of mild cognitive impairment. J Alzheimer’s Dis.

[34] Arlati S, Greci L, Mondellini M, Perego P, Rahmani AM, TaheriNejad N (2018). A virtual reality-based physical and cognitive training system aimed at preventing symptoms of dementia. Wireless mobile communication and healthcare.

[35] Dascal J, Reid M, Ishak WW (2017). Virtual reality and medical inpatients: a systematic review of randomized controlled trials. Innov Clin Neurosci.

[36] Kim O, Pang Y, Kim J (2019). The effectiveness of virtual reality for people with mild cognitive impairment or dementia : a meta-analysis. BMC Psychiatry.

[37] Huygelier H, Schraepen B, van Ee R, Vanden Abeele V, Gillebert CR (2019). Acceptance of immersive head-mounted virtual reality in older adults. Sci Rep.

[38] Ali Mirza R, Yaqoob I (2018). Effects of combined aerobic and virtual reality-based cognitive training on 76 years old diabetic male with mild cognitive impairment. J Coll Physicians Surg Pak.

[39] Bapka V, Bika I, Kavouras C, Auer ME, Tsiatsos T (2018). Brain plasticity in older adults: could it be better enhanced by cognitive training via an adaptation of the virtual reality platform FitForAll or via a commercial video game?. Interactive mobile communication, technologies and learning.

[40] Mrakic-Sposta S, Di Santo SG, Franchini F (2018). Effects of combined physical and cognitive virtual reality-based training on cognitive impairment and oxidative stress in MCI patients: a pilot study. Front Aging Neurosci.

[41] Folstein MF, Folstein SE, Fanjiang G (2009). MINIMENTAL (MMSE)—Przewodnik Kliniczny.

[42] Hodges JR, Larner AJ (2017). Addenbrooke’s cognitive examinations: ACE, ACE-R, ACE-III, ACEapp, and M-ACE. Cognitive screening instruments.

[43] Brzeziński J (1947). Wechsler D (1896–1981). Skala Inteligencji D. Wechslera Dla Dorosłych : Wersja Zrewidowana - Renormalizacja WAIS-R (PL) : Podręcznik. Warszawa: Pracownia Testów Psychologicznych; 2007.

[44] D’Elia LF, Satz P, Uchiyama CL, White T (2012). Kolorowy Test Połączeń CTT: Podręcznik Dla Specjalistów.

[45] Brickenkamp R, Dajek ER, Tucholski P (2003). Test D2: Test Badania Uwagi: Podręcznik.

[46] Sivan AB (1996). BENTON (BVRT)—Podręcznik Oryginalny.

[47] Lezak MD, Howieson DB, Bigler ED, Tranel D (2012). Neuropsychological assessment.

[48] Fritz CO, Morris PE, Richler JJ (2012). Effect size estimates: current use, calculations, and interpretation. J Exp Psychol Gen.

