# Supplementary material for: Effects of virtual reality-based cognitive training in older adults living without and with mild dementia: a pretest–posttest design pilot study
Source: BMC Res Notes. 2019 Nov 27;12:776. doi: 10.1186/s13104-019-4810-2 (PMC6882084; doi:10.1186/s13104-019-4810-2)
Supplement: Supplementary file 1 — Additional file 1: Figure S1. The structure of the intervention. [file 13104_2019_4810_MOESM1_ESM.docx]

Screening tests

Cognitive measures

M

+

A

L

+

V

M

+

A

L

+

V

M

+

A

L

+

V

M

+

A

L

+

V

Cognitive measures

**PRETEST**

**INTERVENTION: 8 training sessions**

**POSTTEST**

4 weeks

2-3 days

2-3 days
